# Impact of host immunity on HTLV-1 pathogenesis: potential of Tax-targeted immunotherapy against ATL

**DOI:** 10.1186/s12977-019-0484-z

**Published:** 2019-08-22

**Authors:** Mari Kannagi, Atsuhiko Hasegawa, Yoshiko Nagano, Shuichi Kimpara, Youko Suehiro

**Affiliations:** 1 Department of Immunotherapeutics, Graduate School of Medical and Dental Sciences, Tokyo Medical and Dental University, 1-5-45 Yushima, Bunkyo-ku, Tokyo, 113-8519 Japan; 2 Department of Immunology, Graduate School of Medical and Dental Sciences, Tokyo Medical and Dental University, Tokyo, Japan; 3 Department of Hematology, National Kyushu Cancer Center, Fukuoka, Japan

**Keywords:** HTLV-1, ATL, Innate immunity, Acquired immunity, Interferon, IL-10, Tax, CTL, Dendritic cell, Tumor vaccine

## Abstract

Human T-cell leukemia virus type-1 (HTLV-1) causes adult T-cell leukemia/lymphoma (ATL), HTLV-1-associated myelopathy/tropical spastic paraparesis (HAM/TSP), and other inflammatory diseases. There is no disease-specific difference in viral strains, and it is unclear how HTLV-1 causes such different diseases manifesting as lymphoproliferation or inflammation. Although some progress has been made in therapies for these diseases, the prognosis for ATL is still dismal and HAM/TSP remains an intractable disease. So far, two regulatory proteins of HTLV-1, Tax and HBZ, have been well studied and shown to have pleiotropic functions implicated in viral pathogenesis. Tax in particular can strongly activate NFκB, which is constitutively activated in HTLV-1-infected cells and considered to contribute to both oncogenesis and inflammation. However, the expression level of Tax is very low in vivo, leading to confusion in understanding its role in viral pathogenesis. A series of studies using IL-2-dependent HTLV-1-infected cells indicated that IL-10, an anti-inflammatory/immune suppressive cytokine, could induce a proliferative phenotype in HTLV-1-infected cells. In addition, type I interferon (IFN) suppresses HTLV-1 expression in a reversible manner. These findings suggest involvement of host innate immunity in the switch between lymphoproliferative and inflammatory diseases as well as the regulation of HTLV-1 expression. Innate immune responses also affect another important host determinant, Tax-specific cytotoxic T lymphocytes (CTLs), which are impaired in ATL patients, while activated in HAM/TSP patients. Activation of Tax-specific CTLs in ATL patients after hematopoietic stem cell transplantation indicates Tax expression and its fluctuation in vivo. A recently developed anti-ATL therapeutic vaccine, consisting of Tax peptide-pulsed dendritic cells, induced Tax-specific CTL responses in ATL patients and exhibited favorable clinical outcomes, unless Tax-defective ATL clones emerged. These findings support the significance of Tax in HTLV-1 pathogenesis, at least in part, and encourage Tax-targeted immunotherapy in ATL. Host innate and acquired immune responses induce host microenvironments that modify HTLV-1-encoded pathogenesis and establish a complicated network for development of diseases in HTLV-1 infection. Both host and viral factors should be taken into consideration in development of therapeutic and prophylactic strategies in HTLV-1 infection.

## Background

Human T-cell leukemia virus type-1 (HTLV-1) causes two distinct diseases, adult T-cell leukemia/lymphoma (ATL) in approximately 4% and HTLV-1-associated myelopathy/tropical spastic paraparesis (HAM/TSP) in less than 2% of infected individuals [1–3]. ATL is a malignant lymphoproliferative disease with poor prognosis [4, 5], while HAM/TSP is a chronic inflammatory disease of the spinal cord [6, 7]. HTLV-1 also causes other inflammatory diseases such as uveitis and pulmonary diseases [8, 9]. However, as yet, the mechanisms by which HTLV-1 causes such a variety of diseases are not well understood.

NFκB is constitutively activated in HTLV-1-infected cells and implicated in both tumorigenesis and inflammation [10]. Among HTLV-1 gene products, HTLV-1 Tax and HTLV-1 basic leucine zipper factor (HBZ) are multifunctional and supposedly contribute to viral pathogenesis [11–14]. Transgenic mice expressing Tax or HBZ proteins exhibit both tumors and inflammation, supporting this notion [15–17]. Tax, encoded by sense mRNA, activates NFκB, CREB, AP-1, and NF-AT, among others, up-regulating various host genes related to cell activation and proliferation [12]. HBZ is encoded by the antisense HTLV-1 genome and activates TGF-β/Smad pathway, promoting FOXP3 expression, but suppresses CREB, AP-1, NF-AT and classical NFκB pathways, competing with Tax functions [18]. Other HTLV-1 accessory proteins including p12, p8, p30 and p13 potentially contribute to viral persistence by degradation of MHC-I, alteration of T-cell receptor signaling, and suppression of Tax expression [19].

Despite its oncogenic potential, HTLV-1 Tax protein is undetectable by serological means in freshly isolated peripheral blood mononuclear cells (PBMCs) from HTLV-1-infected individuals, while it is rapidly induced in ex vivo culture [20]. Because Tax is required for trans-activating other HTLV-1 genes encoding structural proteins, these viral proteins are also undetectable in primary PBMCs. However, HTLV-1 gene expression in vivo is not silent because most HTLV-1 infected individuals maintain antibodies to HTLV-1 structural proteins. In PBMCs of HTLV-1-infected individuals, the sense mRNA of the HTLV-1 genome is barely detectable while HBZ mRNA is continuously detectable by RT-PCR [14, 21]. Furthermore, a recent report indicated the presence of small amounts of HBZ protein in primary ATL cells [22].

Contrary to the expression levels of Tax and HBZ, the frequency of Tax-specific cytotoxic T lymphocytes (CTLs) is greater than that of HBZ-specific CTLs [23]. Tax is a major target antigen for HTLV-1-specific CTLs [24, 25], and the Tax-specific CTL response is conserved in many HTLV-1-carriers, while impaired in ATL patients [26]. Tax-specific CTLs exhibit anti-tumor effects in animal models of HTLV-1-infected lymphoma, suggesting that impaired CTL responses may favor ATL development [27]. We recently developed a therapeutic vaccine to activate Tax-specific CTLs by using Tax peptide-pulsed dendritic cells [28]. A pilot clinical study of this therapeutic vaccine showed favorable clinical outcomes in ATL patients, again raising the question of Tax expression in vivo.

It has been four decades since the discovery of HTLV-1 [29, 30], but there remains confusion in understanding HTLV-1 expression and pathogenesis. In this review, we focus on host immunity, which might be a key factor for both viral expression and pathogenesis, and discuss the following questions to which answers have been long sought after in HTLV-1 research:

1. Why does HTLV-1 cause two distinct types of disease?
2. How is HTLV-1 expression suppressed in vivo and induced in vitro?
3. Can Tax-targeted vaccines produce anti-ATL effects?

## How does HTLV-1 cause two distinct types of disease?

HTLV-1 mainly causes two distinct diseases, ATL and HAM/TSP, manifesting as lymphoproliferation and chronic inflammation, respectively. Because there is no apparent disease-specific difference in HTLV-1 strains, host factors and/or other co-factors are likely to be essential for the development of these lymphoproliferative or inflammatory diseases in HTLV-1 infection.

### Differences between ATL and HAM/TSP

The differences between these two diseases are summarized in Table 1. There is a clear difference in sex; incidence of ATL is greater in males, whereas incidence of HAM/TSP is greater in females [3, 31]. The route of transmission also partly differs; ATL develops mainly in the individuals infected through vertical routes, while HAM/TSP develops in both populations infected via vertical and horizontal routes [32]. Some differences in HLA alleles have also been reported [33, 34].

**Table 1:** Difference between ATL and HAM/TSP

	ATL	HAM/TSP
Pathology	Malignant lymphoma/leukemia [4]	Chronic inflammation with demyelination [6, 7]
Age of onset	Varies among geographic areas [128–130]^a^	40–50 years (average) [3]
Sex	Male > female [131]	Male < female [3]
Route of transmission	Mostly vertical [32]	Vertical and horizontal [32, 132]
Tax mRNA (sense)	Low expression [21]	Low expression but higher than ACs [35]
Tax protein	Undetectable in PBMCs but inducible in culture (in about 1/2 cases) [20]	Undetectable in PBMCs but inducible in culture [133]
HBZ mRNA (anti-sense)^b^	Constitutively expressed [14]	Constitutively expressed [36]
HBZ protein	Small amount in the nucleus [37]	Small amount in the cytoplasm [37]
Tax-specific CTL response	Impaired [26]	Activated [24]
Cytokines	Elevated IL-10 in the serum [41]	Elevated proinflammatory cytokines in the serum and CXCL10 in the CSF [38–40]

*ACs* asymptomatic HTLV-1 carriers, *ATL* adult T-cell leukemia, *CSF* cerebrospinal fluid, *CTL* cytotoxic T lymphocyte, *HAM/TSP* HTLV-1 associated myelopathy/tropical spastic paraparesis, *PBMC* peripheral blood mononuclear cell, *y* years

^a^The mean ages of ATL onset reported are 43 y in Jamaica [128], 67.5 years in Japan [129], and 52 years in the United States [130]

^b^Greater amounts of HBZ mRNA in ATL patients, while not significantly different when standardized by proviral loads [36]

HTLV-1 Tax is undetectable at the protein level in PBMCs from patients with either disease, while Tax mRNA levels are slightly higher in HAM/TSP patients than asymptomatic HTLV-1 carriers (ACs) [35]. HBZ mRNA levels in PBMCs are higher in ATL than in HAM/TSP, but the difference is reported to be insignificant when standardized by proviral load [36]. A recent report indicated that the localization of HBZ in infected cells may differ between the diseases, with HBZ being localized to the nucleus in ATL while it is present in the cytoplasm in HAM/TSP [37].

Cytokine profile in the serum also differs between the two diseases. IL-10 levels are elevated in the serum of ATL patients, while pro-inflammatory cytokines and chemokines such as IFNγ, TNFα, CXCL9, and CXCL10 are elevated in HAM/TSP patients [38, 39]. HTLV-1-infected T-cells from HAM/TSP patients potently secrete IFNγ and induce neurotoxic chemokines such as CXCL10 from astrocytes in the central nervous system [40]. In contrast, production of IL-10 [41], or even loss of cytokine production have been reported in ATL cells [42].

For HTLV-1-specific T-cell responses, there is a marked difference between the two diseases. The Tax-specific CTL response is elevated in HAM/TSP patients while impaired in those suffering from ATL [26]. Because these CTLs are supposedly critical for anti-tumor surveillance in HTLV-1 infection, their impairment likely favors leukemogenesis. However, the reason for the differing CTL responses in the two diseases is not well understood, and the immunosuppressive state in ATL patients may at least be involved.

### Mechanisms of immune suppression in ATL patients

In general, ATL patients are under immunosuppressive conditions [43]. This may be partly attributed to IL-10-dominant conditions in ATL patients [41]. Both Tax and HBZ promote IL-10 production [18, 44]. TGF-β production from ATL cells may also contribute to immune suppression. Tax promotes TGF-β production but suppresses TGF-β/Smad signaling in HTLV-1-infected cells [45, 46]. HBZ augments TGF-β/Smad signaling, inducing FOXP3, which is frequently expressed in ATL cells, although HBZ inhibits FOXP3 functions [47].

In addition to generalized immune suppression, ATL patients exhibit impaired HTLV-1-specific T-cell responses, even at earlier stages of the disease, such as smoldering and chronic type ATL. This is not merely a result of generalized immune suppression, as the T-cell response against cytomegalovirus is mostly conserved at early stages [26]. Such antigen-specific T-cell suppression is usually established through immune tolerance and/or T-cell exhaustion. In HTLV-1 infection, both mechanisms are possible.

Because the major route of mother-to-child HTLV-1 infection is breastfeeding from an HTLV-1-infected mother, newborn tolerance and/or oral tolerance may potentially be induced. In a rat model, HTLV-1-specific T-cell tolerance was induced by oral HTLV-1 infection, resulting in elevated proviral load [48]. Epidemiological studies indicate that vertical HTLV-1 infection is one of the risk factors of ATL development [32], which could be partly attributed to impaired HTLV-1-specific CTL responses as a result of immune tolerance. The mechanisms of immune tolerance involve regulatory T cells (Tregs) producing IL-10 and TGF-β, potentially contributing to general immune suppression as well [49].

T-cell exhaustion due to persistent viral infection also causes virus-specific immune suppression by induction of immune checkpoint receptors [50]. Expression of programmed cell death protein-1 (PD-1) on Tax-specific CTL, and functional improvement of these cells by blockade of the PD-1/PD-Ligand 1 (PD-L1) interaction have been reported in ex vivo studies using PBMC from HTLV-1-infected individuals including ATL patients [51, 52]. Genetic alteration of PD-L1 in ATL cells to elevate expression may promote T-cell exhaustion [53]. However, a recent report indicated that the use of PD-1 antibody (Nivolumab) in indolent types of ATL patients resulted in disease progression [54], suggesting an anti-tumor role of the PD-1/PD-L1 pathway at indolent stages of ATL. Another clinical trial of PD-1 antibody for advanced stage ATL patients is underway in Japan [55]. PD-1 expression on HTLV-1-specific CTLs was reported to be lower in HAM/TSP patients when compared with ACs [56], suggesting that the status of T-cell exhaustion in Tax-specific CTLs may differ between the diseases. Interestingly, HBZ induces another immune checkpoint molecule, T cell immunoglobulin and ITIM domain (TIGIT), and interacts with thymocyte expressed molecule involved in selection (THEMIS) that binds to SHP-2, resulting in inhibition of the checkpoint functions and localization of HBZ itself in the cytoplasm [57, 58].

### IL-10-mediated signals as a switch toward leukemogenesis

**Fig. 1:**
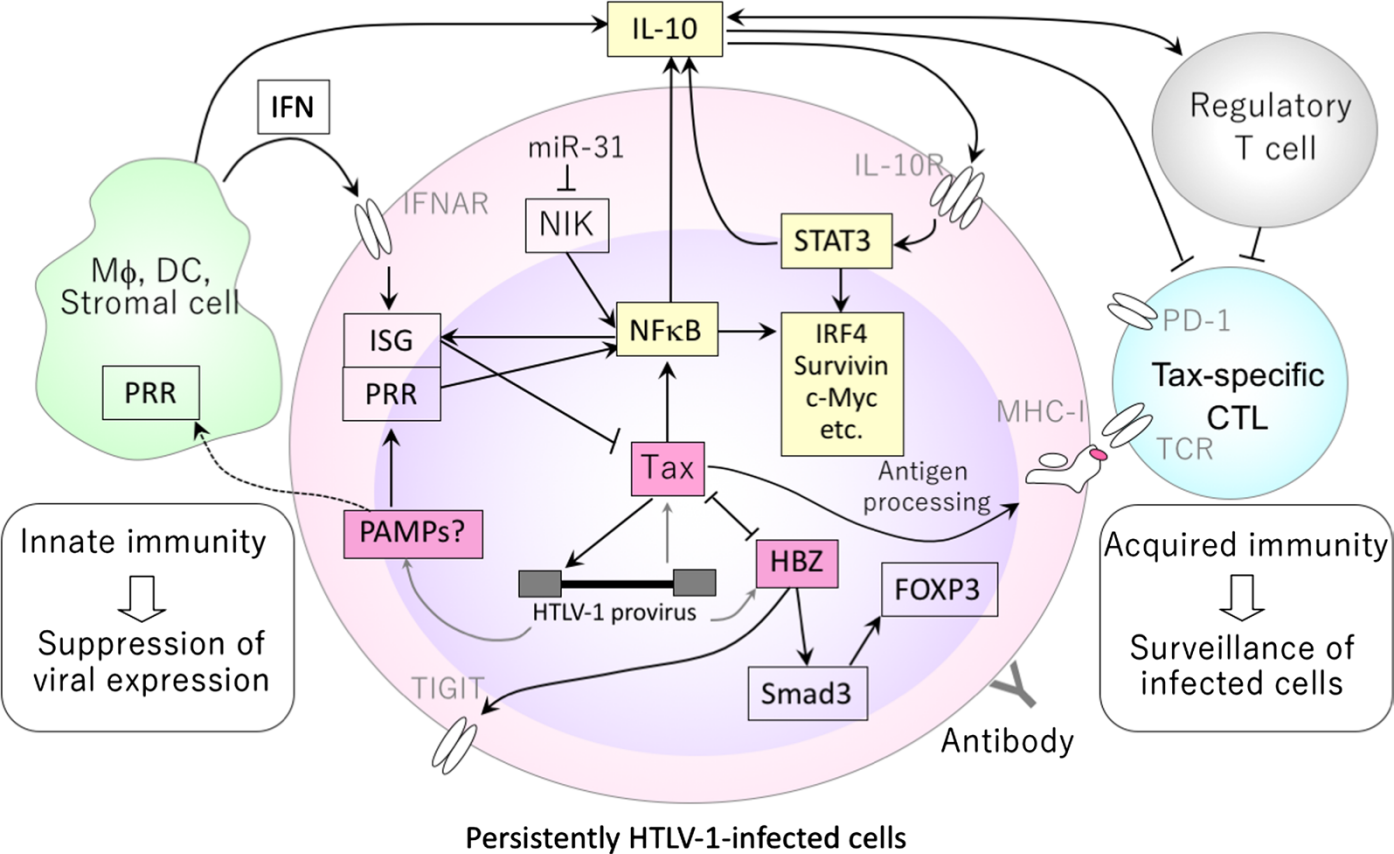
Potential relationship between innate and acquired immunity in HTLV-1 infection (under IL-10-dominant conditions). The hypothesis describing the possible interaction between persistently HTLV-1-infected cells and host immunity is schematically shown. Although HTLV-1 Tax has a strong ability to activate NFκB, type-I IFNs can suppress Tax expression through ISG at a post-transcriptional level. Intrinsic PAMPs (such as viral RNA) might activate PRRs that largely overlap with ISG and potentially suppress Tax expression while activating NFκB. The microenvironmental cytokine balance could be one of the determinants to polarize the feature of HTLV-1-infected cells towards either proliferation or inflammation. This schematic shows the situation where IL-10 dominates. NFκB together with IL-10-mediated STAT3 positive feedback loop induces IRF4, etc. to promote cell proliferation. For acquired immunity, because of the scarcity of Tax expression in vivo, Tax-specific CTLs may eliminate only a limited number of HTLV-1 infected cells, but still contribute to immune surveillance. However, this surveillance becomes increasingly insufficient when CTL function is impaired by immune tolerance and T-cell exhaustion, which may be accelerated by IL-10, TGF-β, IFNs, and Tregs

While IL-10 is known to suppress inflammation and T-cell immune responses [59], our recent study indicated that IL-10 may play a more active role in leukemogenesis by promoting proliferation of HTLV-1-infected cells [60]. Exogenously added IL-10 converted the otherwise slow-growing IL-2-dependent HTLV-1-infected T-cell lines derived from HAM/TSP patients into fast-growing ones. This was associated with phosphorylation of STAT3 and induction of survivin, IRF4, and IL-10, all of which are characteristic of ATL cells. This finding strongly suggests that auto- or paracrine IL-10 produced by the HTLV-1-infected cell and/or the surrounding microenvironment might trigger positive feedback regulation of cell proliferation and switch the fate of HTLV-1-infected cells towards leukemogenesis (Fig. 1).

Recently, Kagdi, et al. reported that IL-10 is produced by only a small fraction of HTLV-1-infected PBMCs but not by the majority of ATL cells, resulting in elevated IL-10 levels in the sera in ATL patients [42]. These data suggest that the source of IL-10 production may not necessarily be ATL cells themselves but could be non-malignant HTLV-1-infected cells. A wide variety of HTLV-1-uninfected cells including helper T cells, Tregs, monocytes, macrophages, and dendritic cells can also be the source of IL-10 production.

As an environmental factor, *Strongyloides stercoralis* infection is often associated with ATL [61, 62]. It is intriguing that *S. stercoralis* infection induces IL-10 and TGF-β [63]. This persistent IL-10-predominated cytokine skewing in vivo produced by *S. stercoralis* infection might be a risk factor for ATL development. The microbiome could also be an important determinant for immunological status and worthy of investigation in HTLV-1-infected individuals to find any association with risk of disease development.

Prolonged IFN activation by persistent viral infection can produce an IL-10-predominant cytokine imbalance in the host, which contributes to T-cell exhaustion through induction of PD-1 expression, as shown in the lymphocytic choriomeningitis virus-infected mouse model [64, 65]. Similarly, persistent HTLV-1 infection itself might potentially cause IL-10 predominant conditions.

Genetic factors also potentially influence IL-10 production in persistent viral infections. In HIV-1 infection, single nucleotide polymorphism (SNP) of the IL-10 promoter (-592A) has been implicated in increased susceptibility to HIV-1 infection and AIDS progression [66]. In HTLV-1 infection, a previous report indicated that the -592A allele of the IL-10 promoter was associated with lower HTLV-1 proviral loads and lower Tax-mediated IL-10 transcription than -592C [67]. Association of the -592A allele with lower susceptibility to HAM/TSP has also been suggested in individuals from Kagoshima, Japan [67], although it was not significant in Brazilian population [68].

## What suppresses HTLV-1 expression in vivo?

### HTLV-1 expression in vivo and in vitro

**Fig. 2:**
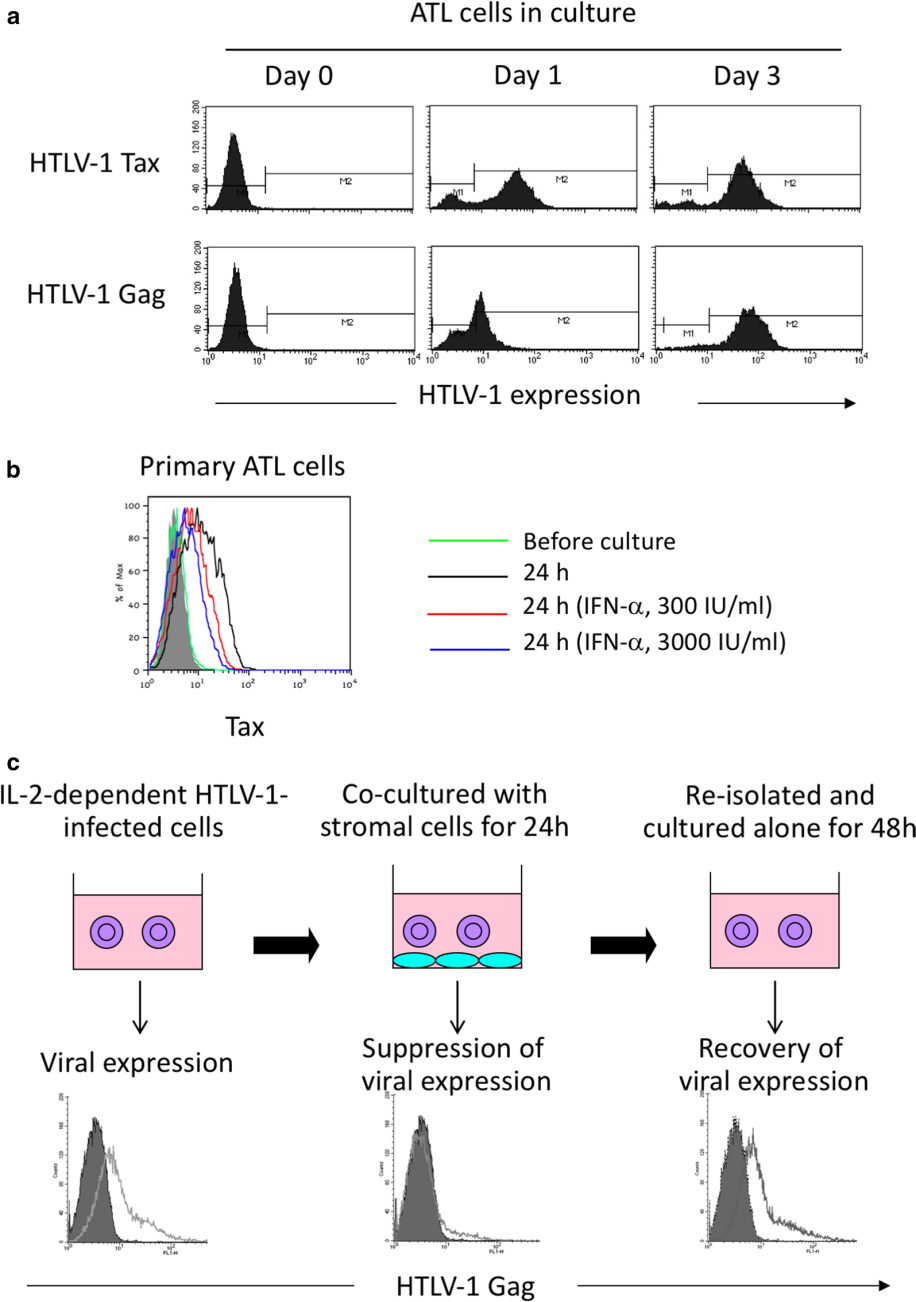
Switch of HTLV-1 expression in vivo and in vitro. **a** HTLV-1 antigens in primary ATL cells isolated from the peripheral blood are undetectable before culture (day 0), but are induced after in vitro culture (day 1 and 3) [20]. **b** The spontaneous induction of HTLV-1 expression in primary ATL cells in the first 24 h of culture is suppressed by exogenously added IFNα [81]. **c** Transient suppression and re-induction of HTLV-1 expression can be reproduced in vitro using IL-2-dependent HTLV-1-infected cells derived from ATL patients. When the HTLV-1-infected cells (purple) are co-cultured with stromal cells (blue), the viral expression becomes undetectable in 24 h but recovers again when the infected cells are re-isolated and cultured alone over the next 48 h [83]

The fact that most HTLV-1-infected individuals maintain HTLV-1-specific antibodies implies the presence of HTLV-1 antigens in vivo. However, HTLV-1 Tax and structural proteins are not detectable in freshly isolated PBMCs from HTLV-1-infected individuals. One of the most peculiar phenomena in HTLV-1 expression is the rapid induction of HTLV-1 expression in PBMCs from HTLV-1-infected individuals in short-term culture, in which Tax becomes detectable first, followed by expression of other structural proteins (Fig. 2a) [20]. HTLV-1 mRNA also rapidly increases within several hours in culture [69].

Such spontaneous induction of HTLV-1 expression in culture of primary PBMCs is observed in asymptomatic HTLV-1 carriers, HAM/TSP patients and about half of ATL patients. In the remaining ATL cases, the ATL cells do not express Tax even after culture because of genetic alterations and epigenetic silencing of HTLV-1 gene expression [70].

The mechanism of the on/off switch of HTLV-1 Tax expression in vitro and in vivo is not well understood. Requirement of TORC family proteins in long terminal repeat (LTR)-driven Tax expression [71] and decreased expression of TORC2 in vivo have been reported [72]. Positive regulation of Tax expression may involve stress-induced responses, as hypoxia enhanced HTLV-1 transcription [73], and p38 MAPK was up-regulated in primary HTLV-1-infected cells from HAM/TSP patients during spontaneous viral induction at primary culture [74]. Involvement of p38 MAPK was also shown in IL-2-dependent HTLV-1-infected cell lines derived from ATL patients, in which removal of IL-2 resulted in activation of p38 MAPK and up-regulation of HTLV-1 expression [75]. Conversely, inhibition of glycolysis and the mitochondrial electron transport chain reduced the induction of HTLV-1 expression [73].

Negative regulation of HTLV-1 expression may involve type-I IFN responses. Although HTLV-1-transformed cells such as MT-2 cells are known to be resistant to type I IFN [76], IFNα suppresses HTLV-1 expression in IL-2-dependent HTLV-1-infected cell lines, presumably because of much lower Tax expression in these cells compared with MT-2 cells. It is well established that Tax inhibits IFN signals by several mechanisms including inhibition of JAK/STAT signaling [77], competition of CBP/p300 [78], induction of SOCS1 [79] and inhibition of IRF3 phosphorylation [80]. Because there are no detectable levels of Tax in PBMCs of HTLV-1-infected individuals, PBMCs likely retain susceptibility to IFNs. Indeed, IFNα suppresses the spontaneous viral induction in primary ATL cells following ex vivo culture (Fig. 2b) [81]. IFNα also suppresses de novo HTLV-1 infection [82].

Furthermore, when IL-2-dependent HTLV-1-infected cells are co-cultured with stromal cells, viral expression becomes undetectable, yet recovers again when the infected cells are re-isolated and cultured alone (Fig. 2c) [83]. This resembles the spontaneous induction of HTLV-1 expression in primary ATL cells following culture ex vivo. Stromal cell-mediated suppression of HTLV-1 expression involves type I IFN, as the suppression can be abrogated by blockade of the IFNα/β receptor [83].

### IFN signature in HTLV-1-infected cells

Despite potential involvement of type I IFN in suppression of HTLV-1 expression, there is no direct evidence of IFN production in vivo in HTLV-1-infected individuals. This might be partly because type-I IFN works at picomolar concentrations [84]. As HTLV-1 infects mainly via cell–cell contact, IFN responses might occur as a local event. Not only cell-free but also cell–cell HTLV-1 infection can induce type-I IFN production in plasmacytoid dendritic cell (pDC) [85]. Similarly, cell–cell contact of the Dengue or Chikungunya virus-infected cells with pDC induces IRF7 activation and type-I IFN production without inflammatory cytokine responses [86]. Infection of these viruses in the mice genetically engineered to exert pDC-restricted IFN response resulted in induction of interferon stimulated genes (ISGs) in pDCs and control of the viruses even in the absence of systemic type I IFN production, supporting the notion of a local IFN response [86].

Although systemic type I IFN responses are apparently absent in HTLV-1-infected individuals, HTLV-1-infected cell lines and ATL cells express elevated levels of ISGs such as 2′,5′-oligoadenylate synthetase, IP-10, and RNA-dependent protein kinase (PKR) [87, 88]. An IFN signaling signature is also observed in HAM/TSP patients [89]. Forced expression of Tax by a retroviral vector can induce these ISGs in vitro mainly through NFκB activation [87]. However, fresh HTLV-1-infected PBMC do not express detectable levels of Tax. It is unclear whether such marginal levels of Tax can induce NFκB and ISG. Furthermore, NFκB is activated even in Tax-negative ATL cells [10]. Therefore, there must be alternative mechanisms of NFκB and ISG activation, besides Tax.

Constitutive NFκB activation in Tax-negative ATL cells is associated with increased NF-κB-inducing kinase (NIK) [90]. Downregulation of miR-31 by polycomb proteins has been shown to increase NIK, leading to NFκB activation in ATL cells [91]. In advanced stages of ATL, genetic alterations in T-cell receptor signaling pathway mediators in ATL cells may also be involved in the mechanisms of NF-κB activation [92].

Another candidate that may mediate activation of NFκB and ISG could be pattern recognition receptors (PRRs) that recognize various pathogen-associated molecular patterns (PAMPs) and potentially mediate NFκB and IRF3 downstream [93]. In de novo infection with cell-free HTLV-1, pDCs or monocytes can be activated to produce IFNα through signaling pathways mediated by TLR7 or STING, presumably recognizing HTLV-1 RNA or reverse transcribed intermediate DNA [94, 95]. However, in persistently HTLV-1-infected T-cells in vivo, production of cell-free HTLV-1 is limited. Primary ATL cells express anti-sense RNAs from the HTLV-1 provirus, which include not only HBZ RNA but also anti-sense RNAs containing the HTLV-1 LTR region that are read through from the cellular flanking region [96]. Although HBZ does not activate NFκB, the latter RNAs might, as knockdown of these anti-sense RNAs reduced NIK expression and NFκB activity in the Tax-negative ATL cell line ED40515(-). A similar effect was obtained by the use of a PKR-inhibitor. The constitutive expression of anti-sense RNAs containing the HTLV-1 LTR region might potentially stimulate PRRs such as PKR to activate NFκB downstream [96]. In support of this notion, a previous report indicated that RNA at the Rex-responsive element in the LTR potentially activates ISG [88].

### Mechanisms of action of AZT/IFNα therapy

Combination therapy with azidothymidine and IFNα (AZT/IFNα) has been used for ATL patients [97, 98] and shows good clinical responses, especially in indolent-type ATL, but is associated with frequent relapses after cessation of treatment [99, 100]. Although AZT alone does not affect cell growth, IFN-α induces cell growth arrest with reduction of Tax expression, and AZT/IFNα induces p53 activation leading to cell apoptosis in IL-2-dependent HTLV-1infected cells [81]. This is consistent with the clinical observation that the AZT/IFNα is effective in ATL cases without p53 mutations [101]. In HTLV-1-transformed cells, the combination of arsenic trioxide and IFN-α also induces cell cycle arrest and apoptosis by degradation of Tax and reversal of NFκB activation [102]. In other malignancies, IFN is known to up-regulate p53 and enhance susceptibility to chemotherapy [103]. ATL cells spontaneously exhibit enhanced levels of p53, while its function is impaired [104, 105]. As Tax has been shown to inhibit p53 signaling [12, 106, 107], IFNα might suppress Tax expression to such a level that it is unable to affect p53 activation in response to AZT incorporation. However, a recent report indicated the involvement of p53 in AZT/IFNα-mediated apoptosis in Tax-negative ATL cell lines or primary ATL cells as well, suggesting the presence of an additional Tax-independent mechanism [108].

## Significance of Tax-targeted therapeutic vaccine in the anti-ATL response

### Anti-tumor effect of Tax-specific CTLs

HTLV-1-specific T-cell responses are observed in most asymptomatic HTLV-1 carriers and HAM/TSP patients, but impaired in ATL patients. Tax is the major target antigen for T-cells [24, 25]. Env is another common target for the T-cell response [109], while HTLV-1 pol, p12, and p13-specific CTLs have also been reported [110, 111]. These proteins are all encoded in the sense strand of the provirus. HBZ, encoded in the anti-sense strand, is constitutively expressed in infected cells and also recognized by CTLs [23]. However, the frequency of HBZ-specific CTLs is much lower than that of Tax-specific CTLs [112]. HBZ-transfected cells but not HTLV-1-infected cells were efficiently killed by experimentally induced HBZ-specific CTLs, suggesting limited HBZ antigen presentation in the infected cells; however the reason for this is unknown [113].

**Fig. 3:**
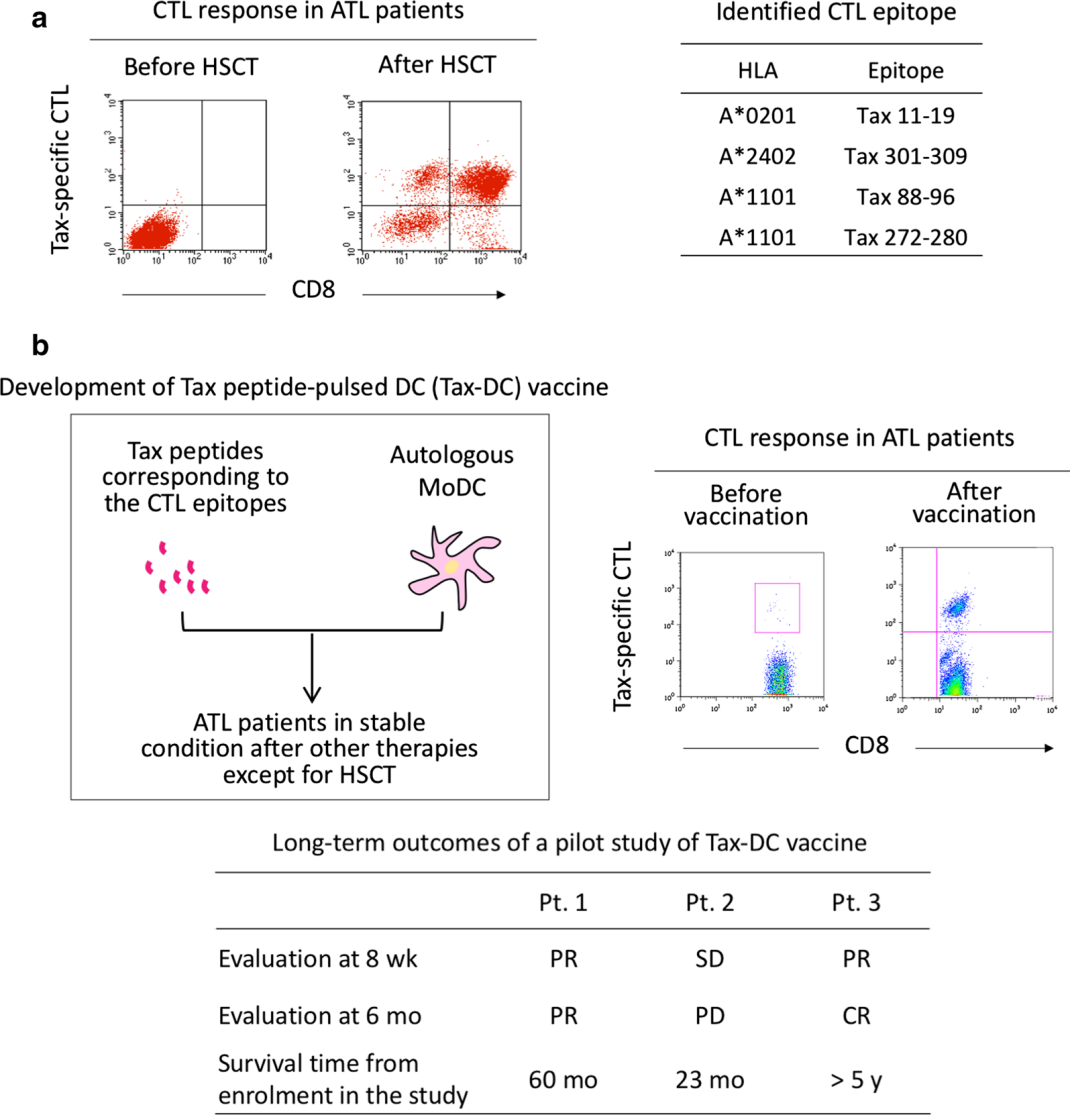
Tax-targeted immunotherapy for ATL patients. **a** The Tax-specific CTL response is mostly impaired in ATL patients but often activated after HSCT and can be evaluated by proliferation of CD8+ Tax-specific CTLs upon stimulation with autologous HTLV-1-infected cells (left). The amino acid sequences of the major epitopes recognized by these Tax-specific CTLs were identified (right) [116, 120]. **b** An anti-ATL therapeutic vaccine (Tax-DC) was developed using autologous DCs pulsed with Tax peptides corresponding to the major epitopes recognized by CTLs (left). Proliferative response of CD8+ Tax-specific CTLs upon stimulation with cognate Tax peptides before and 20 weeks after Tax-DC vaccination in Patient 1 (right), and the clinical outcomes of all three patients in the pilot clinical study (bottom) are shown [28, 119]

Despite Tax expression being undetectable by serological methods in vivo, evidence of Tax antigen presentation in ATL patients was obtained in ATL patients following hematopoietic stem cell transplantation (HSCT). Because frequent relapse is one of the reasons for poor prognosis in ATL patients, HSCT following chemotherapy is recommended in Japan and has been shown to achieve long-term survival in one-third of recipients [114, 115]. In ATL patients who obtained complete remission (CR) after HSCT, activation of the CD8+ Tax-specific CTL response is frequently observed [116]. This is a consequence of a de novo immune response of donor-derived T-cells in the recipient, indicating Tax antigen presentation in vivo (Fig. 3a).

Anti-tumor effects of CD8+ Tax-specific CTLs have been shown in an animal model, in which HTLV-1-infected lymphomas in nude rats were eradicated by adoptive transfer of syngeneic Tax-specific CTLs that had been induced by vaccines using Tax-coding DNA [117] or Tax peptide with a CpG DNA adjuvant [118]. The findings in the ATL patients after HSCT and the animal experiments encouraged development of an anti-ATL Tax-targeted vaccine.

### Tax peptide-pulsed dendritic cell (Tax-DC) vaccine in ATL patients

The first anti-ATL therapeutic vaccine was designed to activate CD8+ Tax-specific CTLs by using Tax peptides as antigen and autologous DCs as adjuvant (Fig. 3b) [28, 119]. The Tax peptides used corresponded to the major epitopes of Tax-specific CTLs restricted to HLA-A*0201, A*2402, and A*1101, which were identified in ATL patients after HSCT [116, 120]. For DC, autologous monocyte-derived DCs were maturated in vitro prior to peptide loading because ATL patients are under immunosuppressive conditions leading to dysfunction of DCs [121].

In the initial pilot study, three ATL patients who were in stable conditions after other therapies except for HSCT were subcutaneously injected with Tax peptide-pulsed autologous DCs three times at fortnightly intervals. All three patients showed clear proliferative responses of Tax-specific CTLs after vaccination without severe adverse effects [28]. Clinical evaluation of the three patients at 6 months was partial response, progressive disease (PD), and CR. In the patient with PD, the lymphoma cells at relapse lacked the ability to express Tax. Survival periods of the three patients after vaccination were 60, 23, and > 60 months. The Tax-DC vaccine is currently under phase I trial, in which an additional three patients have received the Tax-DC vaccine with the same regimen and have maintained CR at least for 2 years after vaccination.

These favorable clinical outcomes of the Tax-DC vaccine indicate the significance of Tax-specific CTLs in maintenance of remission, although they may become ineffective when Tax-negative ATL clones emerge.

### Limitations and prospects of Tax-targeted vaccine

In ATL patients, half of the cases retain the ability to express Tax in ATL cells, while the other half lose this ability [20]. Therefore, the therapeutic effects of Tax-targeted immunotherapy are expected in the former but not in the latter patients. Even in the former cases, innate immune responses suppress Tax expression to various extents in tissues. Therefore, Tax-specific CTLs are presumed to kill only a limited fraction of the HTLV-1-infected cell population. Nevertheless, the results of a clinical study of the Tax-DC vaccine suggested the contribution of Tax-specific CTLs to control ATL cells at least in the cases retaining potential Tax expression. This may be partly because CTLs can recognize a much lower level of Tax antigen than the limit of detection by flow cytometry [122]. Alternatively, the presence of a small number of Tax-positive cells might play a critical role in supporting other HTLV-1 infected cells without Tax expression. The importance of Tax-positive cells has been shown in an ATL-derived MT-1 cell line with only a small percentage of Tax-positive cells, in which knockdown of Tax expression resulted in apoptosis of the whole cell population [123]. A recent study using single cell mRNA FISH visualized Tax mRNA bursts only in a small proportion of cultured clones of the PBMCs isolated from HTLV-1-infected individuals [124].

In ATL patients whose ATL cells lack Tax expression, Tax-specific CTLs cannot directly attack ATL cells. However, these CTLs may still control subdominant HTLV-1-infected cell clones that retain the ability to express Tax, because there are multiple HTLV-1-infected cell clones and the dominant ATL clones sometimes differ among tissues and change during the disease course [125, 126].

Since it takes several weeks to induce immune responses, the Tax-targeted vaccine cannot be the first line therapy for aggressive types of ATL. In the clinical studies, Tax-DC vaccines were administered in ATL patients as a maintenance therapy after chemotherapy. Indolent types of ATL such as smoldering and chronic ATL may be more responsive to the vaccine, as ATL cells likely retain the ability to express Tax more frequently compared with aggressive ATL. Although HAM/TSP patients may also retain the ability to express Tax, HAM/TSP patients usually have active Tax-specific CTLs and less likely benefit from additional vaccines.

Once its safety and efficacy are confirmed, Tax-targeted vaccines might potentially be applied for prophylaxis of ATL development in the future. A small proportion of ACs exhibit insufficient Tax-specific CTL response and elevated proviral load [26], both of which are considered as risk factors of ATL. These ACs may be the possible target population for prophylactic therapy. In a rat model of oral HTLV-1 infection with immune tolerance, Tax-DC vaccine successfully induced Tax-specific CTLs and reduced the HTLV-1 proviral load, suggesting the promising prophylactic potential of Tax-targeting vaccines against ATL [127].

## Conclusion

The complexity of disease mechanisms in HTLV-1 infection results from the host immune responses in concert with HTLV-1-encoded genes (Fig. 1). Although HTLV-1-encoded genes provide multiple mechanisms to activate cells, HTLV-1 infection alone is not sufficient for cell proliferation. IL-10-dominant cytokine skewing could be one of the conditions inducing a proliferative phenotype in HTLV-1-infected cells by stimulating the intrinsic STAT3-IRF4 pathway. Together with its anti-inflammatory/immunosuppressive property, IL-10 signaling may act as a switch between lymphoproliferation and inflammation, driving disease towards ATL development at the early stages.

The extremely low but not silent Tax expression in vivo can be partly explained by type I IFN and ISG, which suppress viral expression mainly at a post-transcriptional level, presumably maintaining HTLV-1 expression at low equilibrium levels in various tissues in vivo. The presence of an IFN signature in HTLV-1-infected cells implies continuous innate immune stimulations going on in these cells, which might also contribute to pathogenesis in HTLV-1 infection.

While Tax protein is undetectable by serological means in vivo, Tax-specific CTLs still seem to recognize HTLV-1-infected cells to some extent. The results of a clinical study of a Tax-targeted therapeutic vaccine in ATL patients indicated a greater impact of Tax-specific CTLs on immune surveillance of HTLV-1 infected cells than expected, again suggesting the presence of Tax expression in vivo. Although further investigation is required, this opens up a new door to early therapy and prophylaxis against ATL.

## Data Availability

Not applicable.

## Abbreviations

AC: asymptomatic HTLV-1 carrier
ATL: adult T-cell leukemia/lymphoma
AZT: azidothymidine
CR: complete remission
CTL: cytotoxic T lymphocyte
DC: dendritic cell
HAM/TSP: HTLV-1-associated myelopathy/tropical spastic paraparesis
HBZ: HTLV-1 basic leucine zipper factor
HSCT: hematopoietic stem cell transplantation
HTLV-1: human T-cell leukemia virus type-1
IFN: interferon
ISG: interferon stimulated gene
LTR: long terminal repeat
NIK: NF-κB-inducing kinase
PAMP: pathogen-associated molecular pattern
PBMC: peripheral blood mononuclear cell
PD: progressive disease
PD-1: programmed cell death protein -1
PD-L1: PD-Ligand 1
PKR: RNA-dependent protein kinase
PRR: pattern recognition receptor
SNP: single nucleotide polymorphism
THEMIS: thymocyte expressed molecule involved in selection
